# Supplementary material for: An analysis and metric of reusable data licensing practices for biomedical resources
Source: PLoS One. 2019 Mar 27;14(3):e0213090. doi: 10.1371/journal.pone.0213090 (PMC6436688; doi:10.1371/journal.pone.0213090)
Supplement: S1 Appendix — (DOCX) [file pone.0213090.s001.docx]

### S1 Appendix. Star criteria for the (Re)usable Data Project

While we try to cover as much of the licensing possibilities of a data resource that we can, in a few special cases we may choose a particular "hat" to wear while evaluating to prevent a combinatorial explosion, which may also reduce the clarity of our curations for the community. In these cases, we may take on the role of a (1) non-commercial (2) academic (3) group that is (4) based in the US and trying to (5) create an aggregating resource, noting that other entities may have different results in the license commentary.

#### A) License is public, discoverable, and standard

**Rationale:** All data sources should publicly state their terms of use in an easy-to-find and unambiguous manner.

- **A.1**) Does the resource have a *single* license?
  - Does the resource’s data area itself (if applicable) have any license information?
    - If yes, take note of where license information is found (is discoverable).
  - Does the resource’s data section have anything license-related in the footer, top-level menu, or "about" page?
    - If yes, note of where license information is found (is discoverable).
  - Does the resource’s homepage have anything license-related in the footer, top-level menu, or ‘about’ page? Please note that while a copyright statement in the footer of the general website may satisfy **A.1**, there are cases where it may not actually be intended to cover the data.
    - If yes, make note of where license information is found (is discoverable).
  - Decision point:
    - Yes, I found a single license: → **HALF STAR** → **A.2**
    - **A.1.1**) No, I found multiple different licenses (i.e. conflicting licenses) and the data cannot be broken easily into multiple individual resources for separate evaluation; the ambiguity here will short circuit further evaluation of the license: → **NO STARS** → **C *and only C***, *no other stars can be awarded in this case*.
    - **A.1.2**) No, I could not find license information in a reasonable location (i.e. license is missing); note that while technically the contents are now covered by default copyright protections in the US, the ambiguity and lack of intent will short circuit further evaluation of the license as clarification and negotiation would be needed: → **NO STARS** → **C *and only C***, *no other stars can be awarded in this case*.

      *Note: If you do NOT find a license or terms using above methods, and happen to find a license using other methods (e.g. via GitHub, Google, etc.), DO make a note of where it was found--we may expand the likely locations in the future. At this point, reasonable discoverability is key for the star, but a found license can still be used when looking at other star criteria. Also note, if you find a single license but it is internally inconsistent, this internal inconsistency is evaluated in* ***B****, not* ***A****.*
- **A.2**) Does the resource use a standard license?
  - Yes, it is as standard license (referenced or copied with only right's holder declaration modifications from a public template), not including public domain declarations: → **add HALF STAR to score from A.1** → **B**
  - **A.2.1**) Is it a public domain declaration? The public domain is a legal concept in many jurisdictions, including the US, rather than a license. While a public domain declaration may work well in some cases, often has very good intent, and could be considered a "standard", ambiguities, the need for legal clarification, and international portability issues make it problematic for our purposes versus a more standard licensing statement. Please see discussions at the Creative Commons and Stanford University Libraries for more background. If yes: → **NO STARS** → **B**
  - **A.2.2**) No, it is a custom or non-standard license: → **NO STARS** → **B**
- Example list of standard licenses; also see license type discussion:
  - Public domain declaration → No, see discussion
  - Any Creative Commons license → Yes
  - Any GNU software or documentation license → Yes, note software license
  - Any BSD or MIT type licenses → Yes, note software license
  - Open Database license → Yes

#### B) License requires no further negotiation and its scope is both unambiguous and covers all of the data

**Rationale:** The licensing terms should be clear for all users without negotiation. If the license allows for non-negotiated reuse, no further legal or human actions should be needed for continuing non-negotiated use of a data resource, except to initially obtain access keys if necessary.

- **B.1**) Does the license clearly define the terms of continuing reuse free of any language that would require human or legal action or negotiation?
  - Yes: → **HALF STAR** → **B.2**
  - No: → **NO STARS** → **B.2**
- **B.2**) Is the scoping of the license complete? To determine this, both of these criteria must be met:
  - **B.2.1**) It is comprehensive: does the license apply to all of the data?
    - Example failures include: “Except where noted…” or “Except where prohibited by the original sources” (e.g. ClinVar, EBI, Monarch).
  - **B.2.2**) It is differentiated: if multiple licenses apply, is it possible to obtain a singly-licensed slice? In the case that a resource holds multiple kinds of content (software, ontologies, or data) or if portions of its records are made available under different licenses, is it possible to obtain a “clean” copy of all of the work that *can* be redistributed without negotiation?
    - Example failures would include: “a reasonable portion of the data may be downloaded and redistributed", etc.
  - Yes (both **B.2.1** and **B.2.2** criteria are met): → **add HALF STAR to score from B.1** → **C**
  - No (fulfills none or just one of the two B2 criteria): → **NO STARS** → **C**
- Examples for **B.1**:
  - Note: requiring registration may be okay, see **C**.
  - License is clearly stated, but requires you to get in touch with the resource’s tech transfer office for a questionnaire → No
  - Language such as: “contact our tech transfer office at xyz@example.com for details” → No
  - Language that requires you to make your data available to the resource into the future outside of licensing → No
- Examples for **B.2**:
  - License applies to partial content, (e.g. onus on user to make determination of the records to which restrictive licensing applies (e.g. ClinVar, EBI, Monarch) → No
  - License provides allowances to a vague subset of the data (e.g. a “reasonable portion” may be redistributed) → No

#### C) The data covered by the license is easily accessible

**Rationale:** License without access is almost meaningless. The data provided by a resource needs to be available to research groups in a transparent and reasonable manner. Any particular content grouping at a resource should be available in its entirety with a single action once reasonable accommodations have been made with the data provider.

- **C.1**) Does the resource provide a “reasonable good-faith location” to access all data groupings with a single "action" (see below for examples of what may constitute an "action"), at an API endpoint or URL?
  - Yes: → **HALF STAR** → **C.2**
  - No: → **NO STARS** → **C.2**
- **C.2**) Does the resource provide a “reasonable and transparent” method of obtaining access to all APIs or URLs outlined above?
  - Yes: → **add HALF STAR to score from C.1** → **D**
  - No: → **NO STARS** → **D**
- Examples “reasonable good-faith location” for data access:
  - URL access over HTTP of entire data set → Yes
  - Dumpable access over API endpoint (e.g. SPARQL dump) → Yes
  - A page with a *small* set of download links for data that will not change in the future → Yes
  - A site where one could easily write a script to scrape the data files in a structured way without cooperation → Yes
  - Set of HTML-only linked pages with data embedded → No
- Examples of “reasonable and transparent” access methods:
  - Unprotected HTTP/S access for an API or downloads → Yes
  - Access by API key for analytics → Yes
  - Access by API key for access control → No
  - Access by API key for access control, but with downloads available allowing timely complete data ingest → Yes
  - Time/size limited access to data that functionally prevents timely complete data ingest → No

#### D) License has little or no restrictions on kinds of (re)use

**Rationale:** Research groups should have the ability to legally access the data to use in their research, build upon and modify it, and publish their results. Ideally, they should be able to do so freely and without encumbrances (except possibly attribution). Forbidding certain kinds of reuse (or explicitly allowing only a narrow kind of reuse) leaves open questions about what prohibited reuse actually constitutes in practice; this determination may require legal consultation. For example, if the provider forbids "editing" the data, is it a prohibition of the license to use just a fraction of the data? Change the format? Build a tool on top of it? Translate it? Because we nevertheless want to recognize any attempt at openness, we award half stars in this category as long as there are redistribution provisions (not just copy/download).

- **D.1**) Are different types of downstream reuse distinguished as allowable or as forbidden?
  - No, all types of reuse are allowed without negotiation, allowing for "reasonable" attribution and redistribution restrictions (e.g. CC BY 4.0 or a similarly permissive license, see license types): → **ONE STAR** → **E**
  - Would a non-legal professional reasonably interpret the license to mean that individuals either in "research” or “non-commercial" contexts could work with the data (add to, modify, build on) and redistribute the results without negotiation *and without requiring the remixed data to have a specific license*?
    - **D.1.1**) Yes: → **HALF STAR** → **E**
    - **D.1.2**) No (e.g. restrictive licenses or all rights reserved, see license types): → **NO STARS** → **E**
- Example restrictions in use and downstream reuse:
  - May not be copied.
  - May not be edited (e.g. invariant text clause).
  - May not be built upon (e.g. "This license forbids derivative works").
  - May not be remixed (i.e. license limitations on how it can be combined with other data sets, such as in the case of the GPL).
  - May not be redistributed under same terms.
  - May be encumbered by patents.
  - May be revocable in some circumstance.
  - May be under an embargo until some date or event.
  - Note that while "personal use only" arguably prohibits many of the above, this is considered separately in **E** as a function of user, rather than use.

#### E) License has little or no restrictions on who can (re)use the data

**Rationale:** When research groups build on and modify data resources, they should be able to make these new products available for redistribution to other researchers in some unencumbered way, giving them the same opportunities to do the same. Ideally, they should be able to pass on their work freely and redistribute it to any party without restriction. Forbidding certain kinds of people or institutions (or explicitly allowing only a narrow definition of such) leaves open questions about what this means; this determination may require legal consultation. For example, if a license forbids commercial use, it may also prohibit remixing, redistribution, or cost recovery by not-for-profit companies. Because we nevertheless want to recognize any attempt at openness, we award half stars in this category as long as there are redistribution provisions that amount to more than "personal use", which is not clear as to whether it permits sharing/remixing within a research group for academic purposes.

- **E.1**) Are different types of person groups or “agents” distinguished?
  - No, all types of person groups or “agents” are allowed without negotiation, allowing for “reasonable” attribution and redistribution restrictions (e.g. CC BY 4.0 or a similarly permissive license, see license types): → **ONE STAR** → **DONE**
  - Would a non-legal professional reasonably interpret the license to mean that individuals either in “research” or “non-commercial” contexts could work with the data (add to, modify, build on) and redistribute the results without negotiation *and without requiring the remixed data to have a specific license*?
    - **E.1.1**) Yes: → **HALF STAR** → **DONE**
    - **E.1.2**) No (e.g. restrictive licenses or all rights reserved, see license types): → **NO STARS** → **DONE**
- Example restrictions on who can (re)use data (a.k.a. discrimination classes); bolded classes are given a half-star exception:
  - **Academic**/non-academic: ½
  - Commercial/**non-commercial**: ½
  - **Clinical**/**non-clinical**: ½
  - Personal/non-personal: 0
  - Profit/non-profit (note: "okay" for non-profit can mean that (re)sale is still permitted): 0
  - Employer-specific: 0
  - Geographical or export limitations: 0
  - Disease-specific / research community-specific: 0
