# Supplementary material for: An analysis and metric of reusable data licensing practices for biomedical resources
Source: PLoS One. 2019 Mar 27;14(3):e0213090. doi: 10.1371/journal.pone.0213090 (PMC6436688; doi:10.1371/journal.pone.0213090)
Supplement: S2 Appendix — (DOCX) [file pone.0213090.s002.docx]

### S2 Appendix. List of all reviewed resources, their categorization, and their license

| **Name** | **License category** | **License used** | **Additional metadata** |
| --- | --- | --- | --- |
| Alliance of Genome Resources (AGR) | copyright | unlicensed | biology, MOD, functional annotation, disease-gene association |
| ArrayExpress | permissive | custom | biology, microarray experiments, functional genomics, high-throughput, microarray, sequencing |
| BGee (data) | copyright | unlicensed | biomedical, x-species, expression data |
| BGee (ontology) | copyleft | GPL 3.0 | biomedical, expression data, ontology |
| BioGRID | permissive | MIT | biology, cross-species, protein-protein interaction |
| BRENDA Tissue Ontology | permissive | custom | biology, ontology, enzyme sources |
| Catalogue of Life | restrictive | custom | biology, custom, biodiversity, distribution, biogeography, taxonomy, ontology |
| ChEMBL | copyleft | CC BY-SA 3.0 | biology, biochemical, bioactive drug-like small molecules |
| Clinical Interpretation of Variants in Cancer (CIViC) | permissive | CC0 1.0 | biomedical, human, cancer, precision medicine, variants, variant disease associations |
| ClinVar | permissive | public domain | biomedical, human, disease-gene association, variant-disease association, variant definitions |
| Comparative Toxicogenomics Database (CTD) | restrictive | custom | biology, x-species, disease-gene association |
| dbGaP (public) | unknown | inconsistent | biology, human, genotype-phenotype |
| DECIPHER | private pool | custom | biology, human, gene, genotype, rare disease, phenotype, variant, submicroscopic chromosomal imbalance, rare sequence variants |
| dictyBase | copyright | unlicensed | biology, MOD, genotype-phenotype association, disease-model association, gene expression |
| DrugBank | restrictive | CC BY-NC 4.0 | pharmacology, drug, bioinformatics, cheminformatics, drugs, drug-protein interactions, targets, pathways |
| DrugCentral | copyleft | CC BY-SA 4.0 | pharmacology, drug-target interaction, chemical structure of drugs, drug, disease |
| Dryad Digital Repository | permissive | CC0 1.0 | general, any, data, literature |
| ENCODE | permissive | custom | biology, genomic resource, genomic elements |
| Fantom5 | permissive | CC BY 4.0 | biology, human, gene expression |
| FlyBase | restrictive | custom | biology, MOD, genotype-phenotype association |
| Genomic Data Commons (GDC) | copyright | unlicensed | biology, human, cancer genome, variants, mRNA and miRNA sequence data |
| Genome Aggregation Database (gnomAD) | copyleft | ODbL 1.0 | biology, human, exome sequencing data, genome sequencing data, disease-specific genetic studies, population genetic studies |
| Gene Ontology (annotations) | permissive | CC BY 4.0 | biology, x-species, gene annotation, gene association, biological process, molecular function, cellular component |
| Gene Ontology (ontology) | permissive | CC BY 4.0 | biology, x-species, ontology, biological process, molecular function, cellular component |
| GTEx | permissive | custom | biology, human, gene expression |
| Human Metabolome Database (HMDB) | restrictive | custom | biology, human, metabolomics, clinical chemistry, biomarkers |
| Human Phenotype Ontology (HPO) | restrictive | custom | biology, human, disease-phenotype association |
| International Mouse Phenotyping Consortium (IMPC) | copyright | all rights reserved | biology, mouse, genotype-phenotype association |
| Kyoto Encyclopedia of Genes and Genomes (KEGG), FTP | restrictive | custom | biology, genomic resource, gene-pathway association, disease-gene association, orthology |
| Mouse Genome Informatics (MGI) | permissive | custom | biology, MOD, genotype-phenotype association, disease-model association, gene expression |
| Monarch Initiative | unknown | inconsistent | biology, x-species, gene, genotype, disease, phenotype, variant, disease-phenotype associations, genotype-phenotype associations |
| Mouse Phenome Database (MPD) | permissive | custom | biology, MOD, genotype (strain)-phenotype association |
| MSigDB | permissive | CC BY 4.0 | biology, gene sets |
| MyGene.info | restrictive | custom | biology, genomic resource, gene definition |
| MyVariant.info | restrictive | custom | biology, genomic resource, variants, variant annotation |
| National Center for Biotechnology Information (Gene) | unknown | inconsistent | biology, genomic resource, gene definition, taxon definition, gene-publication association |
| neXtProt | restrictive | CC BY-ND 3.0 | biology, human, protein-related data, protein functional data, protein-protein interaction, subcellular location |
| Online Mendelian Inheritance in Animals (OMIA) | copyright | all rights reserved | biology, veterinary x-species, gene-disease association |
| Mendelian Inheritance in Man (OMIM) | restrictive | custom | biomedical, human, disease-phenotype association, gene-disease association, variant-disease association |
| OncoKB | restrictive | custom | biology, human, variants, cancer genes, gene expression |
| Orphanet portal for rare diseases and orphan drugs (academic access subset) | restrictive | custom | biomedical, human, disease-gene association, disease-phenotype association, disease classification, clinical metadata, disease epidemiology, orphan drugs, ontology |
| Orphanet portal for rare diseases and orphan drugs (open access subset) | restrictive | CC BY-ND 3.0 | biomedical, human, disease-gene association, disease-phenotype association, disease classification, ontology |
| Protein ANalysis THrough Evolutionary Relationships Classification System (PANTHER) | copyright | all rights reserved | biology, genomic resource, orthology |
| Pfam | permissive | CC0 1.0 | biology, protein families, protein family alignments, HMMs |
| Pharos | unknown | inconsistent | biology, disease, targets, ligands |
| PomBase | copyright | unlicensed | biology, MOD, genotype-phenotype association, disease-model association, gene expression |
| Reactome | permissive | CC BY 4.0 | biology, pathway, pathway data |
| Rat Genome Database (RGD) | permissive | custom | biology, MOD, genotype-phenotype association, disease-model association, gene expression |
| Rhea | permissive | CC BY 4.0 | biology, biochemical, enzymes, metabolic networks, reactions |
| Saccharomyces Genome Database (SGD) | permissive | custom | biology, MOD, genotype-phenotype association, disease-model association, gene expression |
| STRING | permissive | CC BY 4.0 | biology, cross-species, protein-protein interaction, protein families |
| The Arabidopsis Information Resource (TAIR, public) | permissive | CC BY 4.0 | biology, MOD, sequence, gene structure, gene expression, functional annotation |
| UniProt | restrictive | CC BY-ND 3.0 | biology, sequence, protein sequence, protein function |
| WikiPathways | permissive | custom | biology, pathway, disease, micronutrient, nanomaterial, ExDNA, renal genomics, adverse outcomes, regenerative medicine, clinical proteomic tumor analysis |
| WormBase | unknown | inconsistent | biology, model organism genome sequences |
| Zebrafish Information Network (ZFIN) | restrictive | custom | biology, model organism database |
