# Supplementary material for: An analysis and metric of reusable data licensing practices for biomedical resources
Source: PLoS One. 2019 Mar 27;14(3):e0213090. doi: 10.1371/journal.pone.0213090 (PMC6436688; doi:10.1371/journal.pone.0213090)
Supplement: S3 Appendix — (DOCX) [file pone.0213090.s003.docx]

### S3 Appendix. Reference information for commonly used licenses

| **LICENSE** | **REFERENCE** |
| --- | --- |
| all rights reserved *standard US copyright* | https://www.copyright.gov/help/faq/ |
| Creative Commons Attribution 4.0 International *CC BY 4.0* | https://creativecommons.org/licenses/by/4.0/legalcode |
| Creative Commons Attribution-NonCommercial 4.0 *CC BY-NC 4.0* | https://creativecommons.org/licenses/by-nc/4.0/legalcode |
| Creative Commons Attribution-NoDerivatives 3.0 *CC BY-ND 3.0* | https://creativecommons.org/licenses/by-nd/3.0/legalcode |
| Creative Commons Attribution-ShareAlike 3.0 *CC BY-SA 3.0* | https://creativecommons.org/licenses/by-sa/3.0/legalcode |
| Creative Commons Attribution-ShareAlike 4.0 *CC BY-SA 4.0* | https://creativecommons.org/licenses/by-sa/4.0/legalcode |
| Creative Commons Zero 1.0 *CC0 1.0* | https://creativecommons.org/publicdomain/zero/1.0/legalcode |
| GNU General Public License v3.0 *GPL 3.0* | https://www.gnu.org/licenses/gpl-3.0-standalone.html |
| MIT License *MIT* | https://opensource.org/licenses/MIT |
| ODC Open Database License v1.0  *ODbL 1.0* | https://opendatacommons.org/licenses/odbl/1.0/ |
